# French Medical News

**Published:** 1864-02

**Authors:** 


### Art. III.—*French Medical News.*

Paris, March 3, 1863.

Periodical literature has had a striking influence upon the progress of medical science in France. It is carried on with unceasing energy, and employs the time and powers of some of the most intellectual men in Europe. No less than forty journals appear at stated intervals; there are some that are published as often as three times in each week, and they are all fully supported by the reading medical world. "The appetite seems to increase by what it feeds upon." Scarcely is there a person interested in the cultivation of the healing art that does not see at least two periodicals weekly. They are found equally necessary for the student and for the experienced practitioner; and no one can feel that he keeps pace with the march of science if he does not know what is contained in their prolific pages. The journals in Paris are, for the most part, either three times a week or weekly; and whilst some of them have their *specialite*, and are devoted to a single branch of the profession, others embrace all its parts, give outlines of lectures, hospital cases, clinical observations, transactions of the medical societies, and reviews of the works that are likely to excite much interest.

*La Gazette des Hopitaux* makes its appearance three times in each week, under the careful superintendence of Dr. Brochen; it is deservedly a favorite with the medical world, and furnishes a faithful picture of all that is passing. *L'Union Medicale* likewise gives three times in each week a mass of scientific and practical observations; its chief editor, M. Amadee Latour, pupils his task with great care and zeal.

The *Gazette Medicale* is superintended by Dr. Jules Guerin, who offers the result of his experience and his acquaintance with the different branches of knowledge. This is brought out only once a week, as well as the *Gazette Hebdomadaire*, which is much esteemed, and its editor, Dr. Dechambre, is considered as admirably adapted for the task he has undertaken. Dr. Brown-Sequard superintends the *Jomnal de Physiologie*, which appears twice in each month. The *Journal des Connaisances Medicales* is circulated every ten days.

Each branch has its journal. Psychology and mental alienation have their exponents; Dr. Baillairger and Dr. Delaseauve superintend these respective periodicals. Dental Surgery, Ophthalmology, and even Homœopathy find the means of giving publicity, either weekly or monthly, to the subjects to which they are devoted.

The medical societies are occupied in debating upon a question rather relating to the moral duties of the physician than to science. It has undergone not only warm discussion within the walls of the schools, but is filling long columns in the journals. This question is: "Ought a physician to disclose to any individual the malady which calls for his attendance upon a patient." The law in France, as laid down in the *Code Napoleon*, formally forbids a medical man to communicate to any one the professional secrets confided to him. But, may not there be sufficient reason for him, according to his judgment, to commit a breach of this law? A case has been put which has brought into the field some warm advocates for a frank disclosure under certain circumstances on the part of the physician.

A young gentleman had paid his addresses to a lady of delicate health, and had been accepted by the family; it, however, came to the ears of the young lady's father that her betrothed had been for some time under the care of a practitioner of considerable eminence, and he had heard such hints thrown out that he had judged it proper to call upon the physician with the hope of learning from him whether his intended son-in-law had any complaint that might prove injurious to his daughter, who was herself somewhat of an invalid. He was courteously received by the Doctor, who, however, positively refused to give the sightest information as to the state of the youth, his patient. He declared that it would be a breach of confidence which no circumstance could warrant. The father dwelt upon the necessity of arriving at truth, and stated that the approaching marriage of his daughter filled him with anxiety from what he had accidentally heard. He pleaded in vain; not a syllable was to be obtained from which he could glean any shadow of information. The marriage took place and fearful were the results. The young wife was affected with secondary syphilitic symptoms, which, from her ignorance and her delicacy, assumed a most formidable character, she not disclosing her state to anybody. An infected infant is brought into the world; the mother, always delicate, dies, and a tragedy of a most afflicting character supervened, in consequence, as it has been alleged, of the improper silence of the medical man. Arguments on both sides have been heard. At two of the medical societies, the opinion has been given by large majorities that one of the first duties imposed upon the doctor is silence. The oath of Hippocrates to that effect is quoted, and there seems to be a disposition generally to consider that the physician is never warranted to give information as to the state of those consigned to his care. The words of the law which appear in the penal code are: "Physicians and other persons who by profession are depositories of secrets confided to them, and who, unless called on by law, shall reveal such secrets, shall be punished by imprisonment from one to six months, and by fine from 100 to 500 francs."

Dr. Civiale has delivered his annual retrospect of his practice in calculous disease within the last year. He has had 69 cases of stone in the bladder for treatment—66 men, 2 women, and 1 child; 45 of these were private patients, 24 were at the hospital; 61 had stone for the first time, 8 had already been subject to the disease; 58 of these cases were operated upon, 45 by lithotrity; 1 fatal occurred only. There were 8 cases relieved only, 10 were submitted to the ordinary operation, of which 3 were cured, 2 relieved, and 5 died; 3 have been operated upon by joining together the two methods of lithotrity and cutting; of these 2 were cured, the third has irritable bladder; 11 did not undergo any operation. An analysis of this *compte rendu* would occupy more space in the *Medical Times and Gazette* than could be given to it, but it would prove highly interesting; it is to be found in the *Gazette des Hopitaux*, in the number published 27th of the last month.

Syphilographie has had an addition in a memoir by Dr. Amedee, Paris, in which he points out the advantage of filiform setons in suppurating buboes.—*Medical Times and Gazette.*

